# Investigation on the Interaction of Dendritic Core Multi-Shell Nanoparticles with Human Red Blood Cells

**DOI:** 10.3390/nano15030187

**Published:** 2025-01-24

**Authors:** Jakob Krauß, Radostina Georgieva, Miroslav Karabaliev, Moritz Hackmann, Pichayut Rerkshanandana, Saranya Chaiwaree, Ulrich Kalus, Axel Pruß, Yu Xiong, Hans Bäumler

**Affiliations:** 1 Institute of Transfusion Medicine, Charité-Universitätsmedizin Berlin, 10117 Berlin, Germany; jakob.krauss@charite.de (J.K.); radostina.georgieva@charite.de (R.G.); moritz.hackmann@charite.de (M.H.); pichayut.rerkshanandana@charite.de (P.R.); saranya_c@payap.ac.th (S.C.); ulrich.kalus@charite.de (U.K.); axel.pruss@charite.de (A.P.); yu.xiong@charite.de (Y.X.); 2 Department of Medical Physics, Biophysics & Radiology, Medical Faculty, Trakia University, 6000 Stara Zagora, Bulgaria; miroslav.karabaliev@trakia-uni.bg; 3 Department of Pharmaceutical Technology and Biotechnology, Faculty of Pharmacy, Payap University Chiang Mai, Chiang Mai 50000, Thailand

**Keywords:** nanoparticles, erythrocytes, hemocompatibility, dendritic core multi-shell carrier, in vivo carriers

## Abstract

The use of nanoparticles is becoming increasingly apparent in a growing number of medical fields. To exploit the full potential of these particles, it is essential to examine their behavior in the blood and their possible interactions with blood cells. Dendritic core multi-shell DendroSol™ nanoparticles (DS-NPs) are able to penetrate into viable layers of human skin, but nothing is known about their interaction with blood cells. In the present study, we analyze the effect of DS-NPs on red blood cells (RBCs) using confocal laser scanning microscopy (CLSM), flow cytometry, sedimentation rate analysis, spectrophotometry, and hemolysis assays. DS-NPs labeled with Nile red (NR) were added to RBC suspensions and their accumulation in the area of the RBC membranes was demonstrated by CLSM as well as by flow cytometry. In the presence of DS-NPs, the RBCs show an increased sedimentation rate, which also confirms the binding of the NPs to the cells. Interestingly, in the presence of DS-NPs, the RBCs are stabilized against hypotonic hemolysis as well as against the hemolytic action of Triton X-100. This proven anti-hemolytic effect could be utilized to enhance the circulation time of RBCs loaded with drugs for prolonged sustained release and drug delivery with enhanced bioavailability.

## 1. Introduction

Nanomaterials and nanoparticles (NPs) are increasingly attracting the attention of the scientific community. Due to their growing clinical relevance, there has been a rapid increase in the number of publications and regulatory studies on nanomaterials and NPs [1,2,3,4]. Nanomedicines, as defined by the U.S. Food and Drug Administration (FDA) and the European Union (EU), are medical products that are no larger than 100 nm in diameter [5]. As of 2022, over 100 nanomedical products are on the market and over 560 are going through clinical trials [4]. NPs have the potential to provide solutions for therapies that were previously difficult or sometimes even impossible to put into practice or were associated with severe adverse effects. For the following areas, NPs are established: targeted tumor therapy, several skin and eye diseases, immune deficiencies, infections, and nervous system disorders [6,7]. Furthermore, NPs are playing an increasingly important role in diagnostic imaging, drug delivery, and vaccine development [1,2,4,8]. The above-mentioned fields of application exploit the well-known EPR effect (enhanced permeability and retention effect) of NPs, which allows for passive or active accumulation in tumor tissue [9,10,11,12,13]. In addition, various guest molecules or therapeutically active drugs can be delivered in customized doses. This significantly reduces the side effects of many medicines [14].

One of the main problems with the application of NPs is the so-called nanotoxicity, which is due to their extremely high surface-to-mass ratio. This implies a much higher reactivity of the same number of products in nanoparticulate formulations compared to the same products in larger formulations, which can induce stronger side effects [15]. Therefore, biocompatibility is crucial for the medical application of NPs. Another common issue with many NPs is their limited biocompatibility and the length of time they remain in the organism, affecting the efficacy of the treatment. Particle accumulation, penetration, and interactions with the liver, spleen, and immune system can reduce their availability in circulation, resulting in the need for higher doses [16,17,18]. The size and surface characteristics of NPs are important factors influencing their retention time in circulation [19,20,21,22].

NPs that bind to or within the membrane of red blood cells (RBCs) can achieve significantly longer systemic availability [17]. RBCs have a lifespan of 90 to 120 days in the human body [2]. They are biologically compatible, reach nearly all organs, and exist in large numbers, making them ideal carriers for a variety of methods [17,23,24,25,26,27,28]. The use of RBCs as carriers can reduce biocompatibility problems, increase cargo capacity, and slow down the diffusion time of drugs as well as NPs [29,30,31,32]. However, it is essential that the physical properties and functions of the carrier cells are not significantly modified to avoid serious side effects [33]. The morphology of RBCs may be affected by NPs that bind to structural proteins in the membrane [34,35]. Instability, deformation, increased permeability, inflexibility, and even hemolysis by modifying the membrane or inducing oxidative stress could be the consequence [36,37,38,39,40,41,42].

The chemical configuration of DendroSol™ nanoparticles (DS-NPs) is a polar core, a non-polar inner shell, and a hydrophilic outer shell (for details, see Figure 2b). The NP can transport both hydrophilic and lipophilic guest molecules. It can be described as a dendritic core multi-shell nanoparticle. The hydrophilic outer layer enables transdermal application by allowing the DS-NPs to pass through the stratum corneum [43]. This feature may reduce the need for intravenous or oral intake and increase patient compliance. On the other hand, intravenous application is indispensable in many cases where systemic treatment is needed.

The aim of our research presented here was to investigate the interactions of DS-NPs with RBCs. We hypothesized that due to their amphiphilic properties, DS-NPs could attach to the glycocalyx, being incorporated into the membrane as well as penetrating into the cytoplasm of RBCs. We applied Nile red (NR)-labeled DS-NPs (NR-DS-NPs) to visualize the attachment of DS-NPs to RBCs and studied the distribution of the NPs by confocal laser scanning microscopy and flow cytometry. We also investigated the influence of DS-NPs on the stability of the RBC membranes applying hemolytic assays in hypotonic conditions and with the hemolytic agent Triton X-100.

## 2. Materials and Methods

### 2.1. Materials

The following were used: DendroSol™ (DS-NP) (DendroPharm GmbH, Berlin, Germany), phosphate-buffered saline (PBS), (Fisher Scientific GmbH, Fair Lawn, NJ, USA), 0.9% sodium chloride (B. Braun Melsungen AG, Melsungen, Germany), DMSO (Sigma Aldrich Chemie GmbH, Seinheim, Germany), Nile red (Sigma Aldrich Chemie GmbH, Seinheim, Germany), Phagotest^®^ (Celonic Deutschland GmbH & Co. KG, Heidelberg, Germany), Phagoburst^®^ (Celonic Deutschland GmbH & Co. KG, Heidelberg, Germany), RBC lysis solution (BioLegend, Inc., San Diego, CA, USA), arachidonic acid (möLab GmbH, Langenfeld, Germany), APC anti-human CD41 (BioLegend, Inc., San Diego, CA, USA), Alexa Fluor 647 anti-human CD62P (BioLegend, Inc., San Diego, CA, USA), paraformaldehyde (Sigma-Aldrich Chemie GmbH, Munich, Germany). DS-NPs were prepared as a stock solution of 100 mg/mL in 0.9% NaCl.

Blood from 17 healthy volunteer donors anticoagulated with sodium citrate was used for the investigations (Ethics Committee of Charité-Universität Berlin-EA1/110/21).

### 2.2. Size and Zeta-Potential Measurements of DS-NPs

The Zetasizer Nano ZS (Malvern Instruments, Ltd., Worcestershire, UK) was used for particle sizing and zeta-potential measurement. For the size measurement, the DS-NP stock suspension was diluted 1:100 with PBS and analyzed using dynamic light scattering (DLS).

The zeta-potential measurements were performed in a disposable cuvette with integrated gold-plated electrodes. The DS-NP stock suspension was diluted 1:25 times with distilled water or NaCl solution to achieve different conductivities (Table 1).

Both size and zeta-potential measurements were conducted in triplicate after equilibration of the samples at 25 °C for 90 s.

### 2.3. Preparation of DS-NPs Labeled with Nile Red (NR-DS-NPs)

A stock solution of Nile red (NR) was prepared in DMSO at a concentration of 0.5 mg/mL. The labeling of the DS-NPs with NR was performed in PBS at room temperature and final concentrations of 10 mg/mL (DS-NPs) and 0.01 mg/mL (NR), respectively. For comparison, NR was added at the same concentration to pure PBS.

The absorption spectra of NR in DMSO, NR in PBS, and NR with DS-NPs in PBS were collected from 250 to 750 nm using the Cytation™ 3 Imaging Reader (BioTek Instruments, Inc., Winooski, VT, USA).

### 2.4. Flow Cytometry and Confocal Laser Scanning Microscopy (CLSM)

For microscopy and flowcytometry, NR-DS-NPs and NR in PBS were prepared at 10 times higher concentrations than as for the spectrophotometry described above. The erythrocytes were isolated by centrifugation (3000× *g*, 5 min, room temperature) (Hettich Type 1705, Andreas Hettich GmbH & Co. KG, Tuttlingen, Germany), washed 3 times with PBS, and set to a hematocrit of 1% with PBS. Then, 900 µL of this erythrocyte suspension was added to 100 µL of the NR-DS-NPs or to the NR diluted with PBS. From each sample, 30 µL was further diluted with 2 mL PBS and analyzed in the flow cytometer (BD FACS Canto II, BD Biosciences, San Jose, CA, USA).

The same samples were examined by means of a confocal laser scanning microscope (CLSM; Zeiss LSM 510 Meta, Zeiss MicroImaging GmbH, Jena, Germany) with a 100× oil immersion objective (a numerical aperture of 1.3). The images were obtained in transmission and fluorescence mode with fluorescence excitation at 488 nm and a 505 nm long-pass emission filter.

### 2.5. Blood Sedimentation Rate

Six blood samples taken and stored at room temperature were used for the measurements. For each of them, the hematocrit value was determined in triplicate using a small centrifuge (Hettich Mikro 220R, Andreas Hettich GmbH & Co. KG, Tuttlingen, Germany), and part of the blood was diluted with PBS to set a hematocrit of 5%. DS-NPs were added at a concentration of 10 mg/mL per 1% hematocrit resulting in a final concentration of 50 mg/mL DS-NPs in the samples. For the control samples, the volume fraction of the particles was supplemented with PBS. All samples and controls were mixed in the Vortex small shaker (Vortex Genie 2 Digital, Fisher Scientific GmbH, Schwerte, Germany). The blood sedimentation was measured in hematocrit tubes, as the required concentration of particles would not have been achievable in sedimentation tubes. The tubes were set up at the same time to obtain visual comparability of samples and controls. Every 10 min, the height of the erythrocytes in all tubes was measured. The same experiment was also carried out with 3 samples of previously washed erythrocytes. These were also diluted to a hematocrit of 5% and then examined using the same methodology.

### 2.6. Hemolysis Assays

#### 2.6.1. Hypotonic Hemolysis

The experiments involved real-time spectrophotometric measurements performed in situ in a cuvette with a 0.5 mm pathlength. The experiment commenced at a physiological concentration of 150 mM NaCl and 100 μL RBC suspension with 1% hematocrit. The osmolarity was decreased by adding 10 μL portions of distilled water. After each portion, the sample was stirred, and the absorbance spectrum was measured each 15 s until equilibration after some minutes. The hemolysis was determined by using the equilibrium values of the absorbance at 700 nm. At this wavelength, the absorbance is due only to the light scattering from the intact erythrocytes and hence is relevant to the percentage of the remaining intact erythrocytes during the hemolysis. For each dilution step, the value of the absorbance was corrected by the dilution quotient by the added amount of water as this process not only decreases the osmolarity but also the hematocrit of the RBC suspension and, consequently, the measured absorbance.

The degree of hemolysis at each osmolarity is expressed by the quantity “Relative hemolysis” (R.H.), derived by the following equation:

$$R.H.=100\frac{A_{i}-A_{0}}{A_{100}-A_{0}},\;\%$$

Here, *A*_i_ is the corrected absorbance at 700 nm at the corresponding osmolarity. *A*_100_ is the absorbance at 700 nm of the initial suspension at physiological conditions in which 100% of the erythrocytes are intact. *A*_0_ is the corrected absorbance at 700 nm at concentration of 50 mM NaCl, in which the sample is completely hemolyzed, i.e., 0% of the erythrocytes are intact.

The effect of the DS-NPs on the hemolysis in hypotonic conditions is determined in parallel experiments with the same set-up with a starting RBC suspension with the same volume and hematocrit and 80 μg/mL DS-NPs.

#### 2.6.2. Hemolysis with Triton-X100

The experiments were conducted in accordance with the following procedure. The absorbance at 700 nm of the RBC suspensions with hematocrit 0.07% was followed during 2.5 min for stabilization. At minute 2.5, DS-NPs were added at varying final concentrations (from 2.2 to 13.2 µg/mL). At minute 7.5, Triton X-100 was added at the same final concentration of 100 µg/mL. The absorbance at 700 nm was recorded in real time every 15 s until it reached the value of 0, i.e., the hemolysis was complete.

## 3. Results and Discussion

### 3.1. Size and Zeta Potential

The measurement of the zeta potential of the DS-NPs was performed at three different conductivities and delivered values corresponding to electrically neutral or weakly negatively charged particles (Table 1). The almost electroneutral zeta potential together with the amphiphilic structure allows the DS-NPs to interact easily with surfaces and to incorporate small drug molecules independently of their electrical charge.

**Figure 1:**
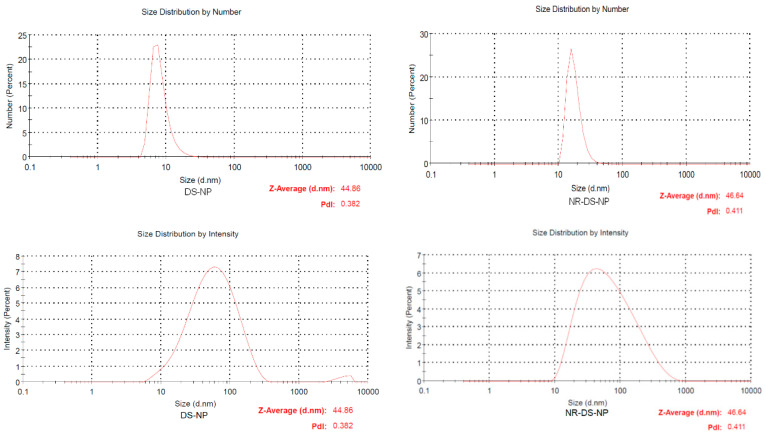
Hydrodynamic diameter of DS-NPs (**left**) and NR-DS-NPs (**right**) determined by dynamic light scattering (DLS) in the number and intensity mode. The measurements were conducted in distilled water at a concentration of 1 mg/mL and 25 °C. (*n* = 3).

The mean hydrodynamic diameter of the DS-NPs was given by the manufacturer with 10 to 20 nm. This is consistent with our own measurements by numbers (Figure 1, upper row). However, if measurements are taken in the intensity mode, there is a clear shift to higher values with a Z-average of 44.9 ± 5.2 nm, and the distribution is much broader. The same applies to the size distributions of the NR-DS-NPs (Figure 1, lower row). The Z-average of the NR-DS-NPs was 46.6 ± 6.1 nm. The DS-NPs and NR-DS-NPs do not differ significantly in size distribution.

### 3.2. Labeling of DS-NPs with NR

**Figure 2:**
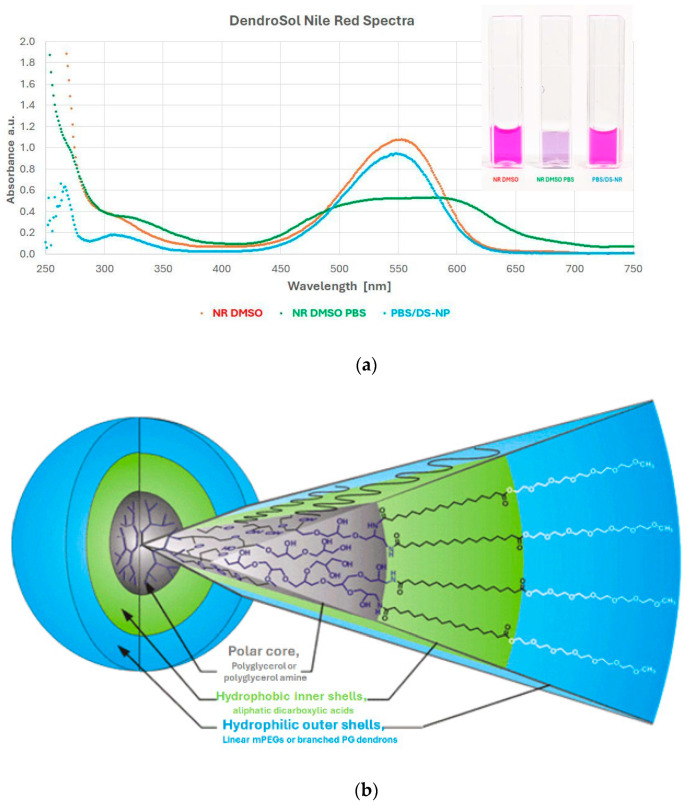
Absorption spectra of NR in different environments (**a**): DMSO (orange curve); PBS/DMSO mixture (green curve); PBS/DS-NP suspension (blue curve). The insert with the cuvettes demonstrates the color change. (**b**) Scheme of DS-NPs (modified from [43]).

NR was used for the fluorescent labeling of the DS-NPs. This dye has almost no fluorescence in aqueous solutions but a strong fluorescence in hydrophobic environments [44]. Solutions of NR in DMSO have a strong pink color; in aqueous environments, the color is blueish. When NR from the DMSO stock solution is added to the DS-NP suspension (in PBS), the color remains pink. In contrast, the addition of the same amount of NR stock solution to pure PBS leads to an immediate change in the color to blueish (insert in Figure 2a).

The described behavior of NR in the different samples is reflected in their spectra. The absorption spectrum of NR in the DS-NP suspension (blue curve) is very close to the absorption spectrum of NR dissolved in pure DMSO (orange curve) (Figure 2). In contrast, in the sample in which PBS was added instead of the particle suspension, a flattening of the absorption spectrum occurs due to the formation of NR aggregates. These effects can only be explained by the incorporation of the dye into the hydrophobic shell of the DS-NP.

### 3.3. Confocal Laser Scanning Microscopy and Flow Cytometery

**Figure 3:**
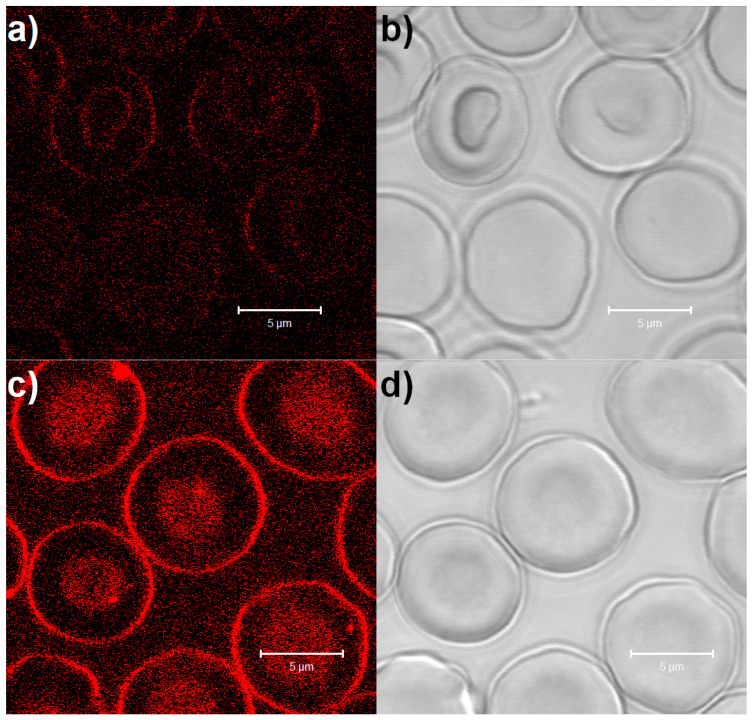
Confocal laser scanning microscopy images of the RBCs incubated with (**a**,**b**) NR dissolved in PBS and (**c**,**d**) DS-NPs preincubated with Nile red. The micrographs are displayed as fluorescence images (**left**: excitation at 488 nm and 505 nm long-pass emission filter) and in the transmission mode (**right**).

To demonstrate that DS-NPs bind to the membranes of the RBCs, we applied NR-DS-NPs. The washed erythrocytes were mixed with solutions of NR in PBS and with NR-DS-NPs and observed using CLSM (Figure 3).

No shape or volume changes could be detected in the transmission mode. However, a clear increase in fluorescence, especially in the peripheral areas of the RBCs, can be seen when the cells were added to the NR-DS-NP solution (Figure 3c). This observation suggests that the DS-NPs interact with the RBC membranes. Due to the multi-shell structure of the DS-NPs, they would be able to attach to either the glycocalyx or the lipid bilayer and/or to the membrane proteins.

**Figure 4:**
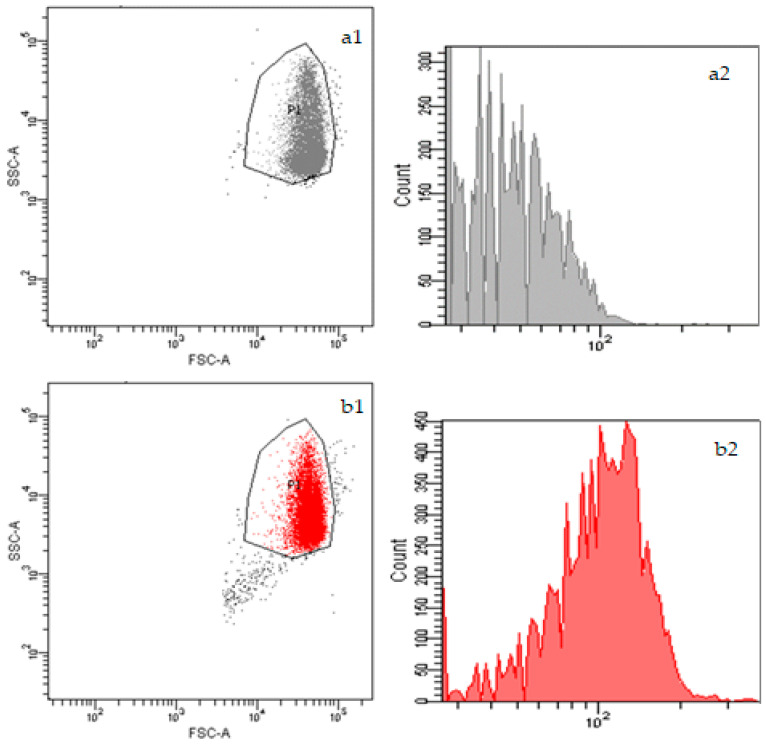
Dot plots and fluorescence intensity histograms of RBC suspensions incubated with (**a1**,**a2**) NR in PBS and (**b1**,**b2**) NR-DS-NPs, respectively. Data are representative of *n* = 2 blood samples.

The RBC suspensions were also analyzed by flow cytometry (Figure 4). The dot plots (Figure 4a1,b1) represent the scattering behavior of the RBCs, which to some extent reflects their shape and volume. It is clearly seen that there are no mentionable differences between the sample with NR in PBS and the one with the NR-DS-NPs, confirming the observation of their similar shape and size by microscopy (Figure 3).

The comparison of the fluorescence intensity histograms of the RBC suspensions with NR in PBS (Figure 4a2) and NR-DS-NPs (Figure 4b2) clearly shows that the addition of NR-DS-NPs results in higher fluorescence intensities in the RBC population. The corresponding mean fluorescence values are also significantly higher in the latter sample (114 (SD: 5.66) for RBCs with NR-DS-NPs and 29 (SD: 5.66) for NR in PBS).

### 3.4. Blood Sedimentation Rate

**Figure 5:**
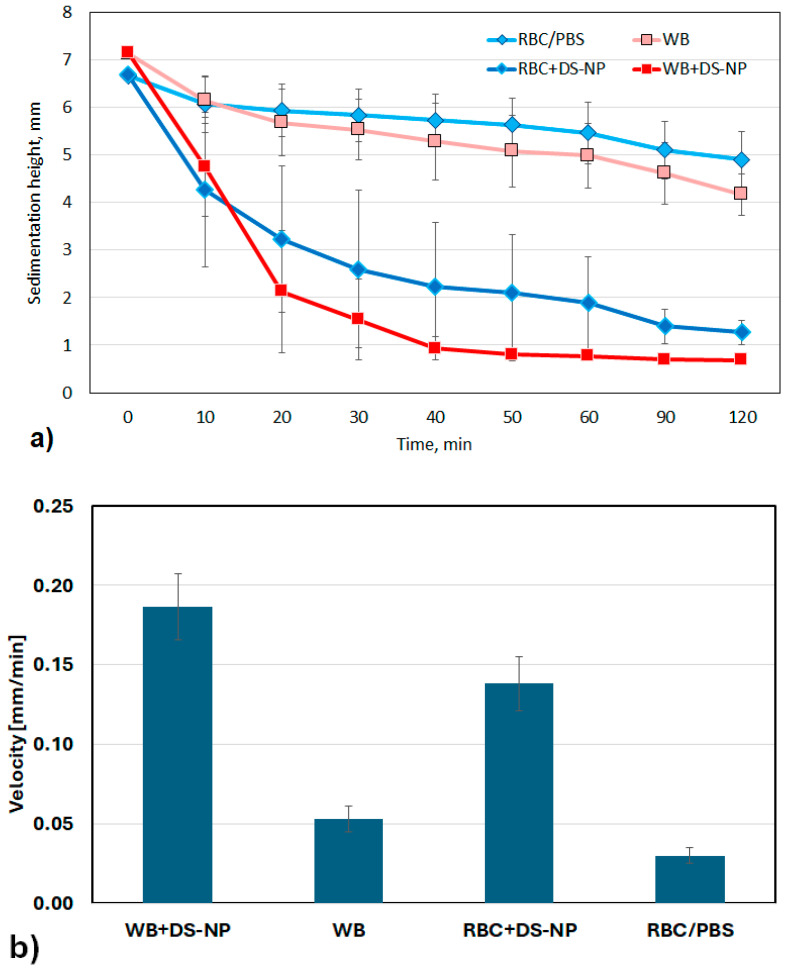
(**a**) Effect of DS-NPs on the sedimentation rate of RBCs determined for whole blood Hct 5% (WB) (*n* = 6) and washed erythrocytes Hct 5% (RBC/PBS) (*n* = 3). (**b**) Sedimentation rates within the first 30 min.

The blood sedimentation rate in all six diluted whole blood (WB) samples spiked with DS-NPs was massively increased compared to the control samples. Even in the three samples with washed RBCs, a clearly faster drop in the erythrocytes in the presence of DS-NPs can be observed (Figure 5a).

The sedimentation rates calculated from the values from 0 to 30 min are shown in Figure 5b. The ratios between the sedimentation velocities with and without DS-NPs, V_(WB+DS-NP)_/V_(WB)_ = 3.50 ± 0.32 or V_(RBC+DS-NP)_/V_(RBC/PBS)_ = 3.19 ± 0.29, do not differ significantly for the WB samples and the samples of washed RBCs. Consequently, it can be assumed that DS-NPs bind to the erythrocytes without causing increased aggregation of the erythrocytes since they only aggregate in the presence of macromolecules in the medium [45].

The density of blood cells ρ can be calculated using the RBC sedimentation rates [46].

ρ_i_ = V_i_/V_k_ (ρ_RBC_ − ρ_k_) + ρ_k_, with i: WB+DS-NP or RBC/PBS and k: plasma or PBS.

Using Stoke’s law to estimate density provides overdetermined values [47]. Here, the law was only used to show that there are no significant differences between the density of RBCs loaded with DS-NPs measured in whole blood and in PBS (Table 2).

### 3.5. Effect of DendroSol™ on Hemolysis of Erythrocytes in Different Conditions

#### 3.5.1. Effect of DendroSol™ NPs on Hemolysis in Hypotonic Conditions

**Figure 6:**
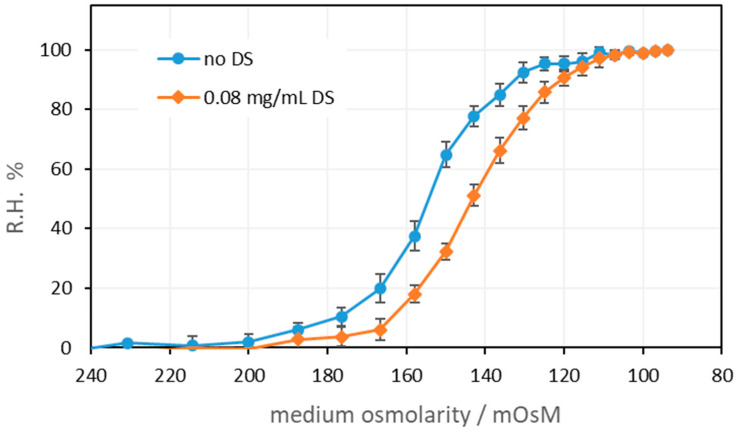
Relative hemolysis in hypotonic conditions with or without DS-NPs. The measurements were performed at a wavelength of 700 nm (*n* = 3).

The results are presented in Figure 6, as a dependence of the relative hemolysis (R.H. %) on the osmolarity of the medium. The relative hemolysis was calculated according to eq. 1 and using the absorbance at 700 nm. As can be seen, the presence of DS-NPs results in the stabilization of erythrocytes in hypotonic conditions. In the presence of DS-NPs, equal values of relative hemolysis are measured for RBC suspensions in NaCl solutions with approximately 10 to 15 mOsM lower osmotic pressure than in the RBC suspensions without DS-NPs (for a DS-NP concentration of 80 µg/mL). In other words, more hypotonic conditions are required in the presence of DS-NPs to cause hemolysis.

#### 3.5.2. Effect of DS-NPs on RBC Hemolysis with Triton-X100

The effect of the non-ionic surfactant Triton X-100 on the RBCs in presence of DS-NPs was investigated using real-time hemolysis assay. Cell lysis with Triton X-100 is a technique commonly used in cell biology for the release and analysis of intracellular compounds. It causes a complete hemolysis of RBCs already at very low concentrations and physiological conditions.

**Figure 7:**
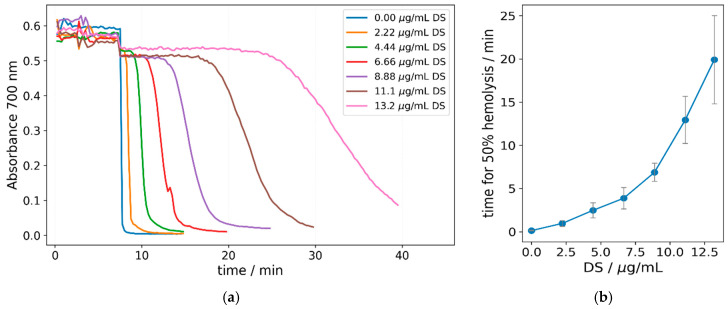
Induced hemolysis by Triton-X100 and different concentrations of DS-NPs. Hemolysis of RBCs induced by Triton-X100 in the presence of different amounts of DS-NPs. (**a**) Representation of the course of hemolysis in real-time using the absorbance of the suspension at 700 nm. (**b**) Dependence of the time for 50% hemolysis caused by Triton-X100 on the concentration of DS-NPs (*n* = 3). In all experiments, the RBC concentration was 0.07% hematocrit and the Triton-X100 concentration was 100 μg/mL (*n* = 3).

The results are shown in Figure 7a. The different curves represent hemolysis in RBC suspensions with the same hematocrit of 0.07% caused by the same amount of Triton X-100 (100 µg/mL) in the presence of different amounts of DS-NPs. As can be seen, the hemolysis is a gradual process that starts after some initial lag period of time and ends after another period of time that is needed for all the RBCs to release their hemoglobin.

The results demonstrate a significant impact of the DS-NPs on the process of hemolysis caused by Triton-X100. With an increasing concentration of DS-NPs in the RBC suspension and a fixed Triton X-100 concentration, the time required for hemolysis increases. In this respect, we chose the time for 50% hemolysis as a parameter for the quantitative characterization of the overall process and membrane stabilization (Figure 7b). According to these results, the stabilizing effect increases exponentially with the concentration of DS-NPs in the RBC suspension. The formation of pores in the lipid membrane of the erythrocytes depends on the Triton-X100 concentration [48]. The DS-NPs appear to be incorporated into the lipid membrane rather than into the glycocalyx of the RBCs, thereby delaying the formation of pores large enough for hemoglobin to pass through the membrane.

## 4. Conclusions

The obtained results in our investigation show clearly that DS-NPs interact with RBCs without any negative effects. The site of interaction is likely the lipid membrane of the RBCs and not the glycocalyx. DS-NPs do not influence the aggregation of RBCs.

Interestingly, in the presence of DS-NPs, the RBCs are stabilized against hypotonic hemolysis as well as against the hemolytic action of Triton X-100. This proven anti-hemolytic effect could be utilized to enhance the circulation time of RBCs loaded with drugs for prolonged sustained release and drug delivery with enhanced bioavailability. Further specific investigations with different drugs, especially with a focus on targeted therapies, will show the entire potential of these highly promising nanoparticles.

## Figures and Tables

**Table 1:** Zeta potential of DS-NPs at different conductivities of the dispersion medium. The values are displayed as Mean ± Standard deviation from 3 or 6 measurements.

Conductivity, mS/cm	Zeta Potential, mV
0.9 ± 0.02 ^1^	−0.7 ± 0.07
2.1 ± 0.17 ^2^	−3.2 ± 0.88
17.4 ± 0.75 ^2^	−4.5 ± 0.35

^1^ *n* = 6; ^2^ *n* = 3.

**Table 2:** Density of RBCs with DS-NPs in whole blood (WB) and in PBS. Density of RBCs without DS-NPs ρ_Ery_ = 1.060 ± 0.015 g/mL, plasma viscosity η_Pl_ = 1.027 ± 0.010 mPa s, η_PBS_ = 1.015 ± 0.010 mPa s. The values are displayed as Mean ± Standard deviation from 3 or 6 measurements.

Sample	Velocity (mm/min)	Density (g/mL)
WB + DS-NP	0.187 ± 0.021	1.1425 ± 0.029
WB	0.053 ± 0.008	
RBC + DS-NP	0.138 ± 0.017	1.1573 ± 0.0231
RBC/PBS	0.043 ± 0.005	

## Data Availability

Data are available after contacting the corresponding author and are stored at the central server of Charité-Universitätsmedizin Berlin.
